# Supplementary material for: Proteomic Profiling of Extracellular Vesicles Isolated from Plasma and Peritoneal Exudate in Mice Induced by Crotalus scutulatus scutulatus Crude Venom and Its Purified Cysteine-Rich Secretory Protein (Css-CRiSP)
Source: Toxins (Basel). 2023 Jul 2;15(7):434. doi: 10.3390/toxins15070434 (PMC10467150; doi:10.3390/toxins15070434)
Supplement: Supplementary file 1 [file toxins-15-00434-s001.zip › Supplementary Table_MS.pdf]

SupplementaryMaterial: Proteomic Profiling of Extracellular Vesicles Isolated from Plasma and Peritoneal Exudate in Mice Induced by *Crotalus scutulatus scutulatus* Crude Venom and Its Purified Cysteine-Rich Secretory Protein (Css-CRiSP)

Armando Reyes, Joseph D. Hatcher, Emelyn Salazar, Jacob Galan, Anton Iliuk, Elda E. Sanchez and Montamas Suntravat

Table S14. List of the top 10 upregulated and downregulated proteins in E-vEV and E-CRiSP-EV samples and their biological processes.

Accession	Gene ID	Biological Functions*
Q6PGF7	Exoc8	This gene has phosphatidylinositol and small GTPase binding activity and plays a role in several biological processes. This includes Golgi to plasma membrane transport, endosome organization, and extracellular matrix disassembly and this protein is predicted to function upstream of or within exocytosis.
P47809	Map2k4	This gene has the following binding activities: ATP, JUN kinase, and mitogen-activated protein kinase. This gene is involved in the positive regulation of smooth muscle cell apoptotic process and acts upstream and within the MAPK cascade, negative regulation of motor neuron apoptotic process, and response to wounding.
P21440	Abcb4	This gene enables the following transporter activities: ATPase-coupled intramembrane lipids, ATPase-coupled transmembrane, and xenobiotic transmembrane (located in plasma membrane).
P09813	Apoa2	This gene encodes a component of high-density lipoproteins (HDL). Mice lacking the encoded protein have low HDL-cholesterol levels, smaller HDL particles, increased clearance of triglyceride-rich lipoproteins, and insulin hypersensitivity.
O08583	Alyref	This gene enables both RNA and single-stranded DNA binding activity which is involved in several processes including: mRNA export from nucleus, regulation of nucleobase-containing compound metabolic process, and viral mRNA export from host cell nucleus. This gene acts upstream within RNA splicing, mRNA processing, and mRNA transport. This gene is part of the part of catalytic step 2 spliceosome, exon-exon junction complex, and transcription export complex.

P17710	Hk1	This gene enables activity such as glucokinase and peptidoglycan binding. It is involved in carbohydrate phosphorylation, positive regulation of cytokine production involved in immune response, and positive regulation of interleukin-1 beta production. Specifically, it is included in the mitochondria.
A0A0B4J1I0	Igkv1-110	This gene is involved in immune response and active in extracellular space.
Q5RKZ7	Mocs1	This gene enables GTP 3',8'-cyclase activity and cyclic pyranopterin monophosphate synthase activity. The gene acts upstream within molybdopterin cofactor biosynthetic processes. Lastly it can be found in cytosol and a part of the molybdopterin synthase complex.
Q9JLI8	Sart3	This gene enables binding activities such as histone, snRNA, and ubiquitin-specific protease. The gene acts upstream and within cell morphogenesis, hematopoietic stem cell proliferation, and homeostasis.
A0A0A6YXN5	Ighv1-43	This gene enables both antigen and immunoglobulin receptor binding activities. Involved in activation of immune response, defense response to other organisms, and phagocytosis as well as the immunoglobulin complex.
Q9DBP5	Cmpk1	This gene enables kinase activities such as CMP, UMP, and nucleoside diphosphate. Commonly located in the nucleolus and predicted to act upstream and within phosphorylation.
Q9JLF6	Tgm1	This Gene enables identical protein binding and protein-glutamine gamma-glutamyl transferase activities. The gene is located in adherens junction and acts upstream as well as within animal organ morphogenesis.
A0A0G2JFE9	Ighv1-76	This gene enables both antigen binding and immunoglobulin receptor binding activities. It is predicted to be involved in including activation of immune response and phagocytosis.
O08689	Mstn	This gene encodes a secreted ligand of the transforming growth factor-ß (TGF-ß) superfamily of proteins. Ligands of this family bind various TGF-ß receptors leading to recruitment and activation of SMAD family transcription factors that regulate gene expression.
P54276	Msh6	This gene enables binding activities such as that of chromatin, damaged DNA, and guanine/thymine mispair. It acts upstream and within several processes including an intrinsic apoptotic signaling pathway. This is in response to DNA damage and somatic diversification of immunoglobulins.
A0A075B5R1	Ighv5-17	This gene enables both antigen and immunoglobulin receptor binding activities. It is involved in several processes such as activation of immune response and phagocytosis.
Q9JIZ0	Cml1	This gene enables cysteine-S-conjugate N-acetyltransferase activity and lysine N-acetyltransferase activity, acting on acetyl phosphate as donor. It is involved in several processes, including negative regulation of apoptotic process, peptide metabolic process, and peptidyl-lysine N6-acetylation. It is located in the mitochondrial inner membrane and can act upstream of or within negative regulation of cell adhesion.
Q9D819	Ppa1	This gene enables pyrophosphatase activity. It is predicted to be involved in phosphate-containing compound metabolic processes.

Q8VHK9	Dhx36	This gene enables activities such as DNA helicase, histone deacetylase binding, and nucleic acid binding. It acts upstream and within response to viruses.
Q505F5	Lrrc47	This gene is expressed in the CNS and enables RNA binding activity and phenylalanine-tRNA ligase activity.
P27046	Man2a1	This gene enables hydrolase activity, hydrolyzing N-glycosyl compounds and mannosidase activity. It acts upstream and within N-glycan processing, animal organ development, and positive regulation of neurogenesis.
P39447	Tjp1	This gene enables both connexin and protein domain specific binding activities. It is involved in the protein localization to cell-cell junction. It is located in the bicellular tight junction.
Q9CW03	Smc3	This gene enables both chromatin and mediator complex binding activities. It acts upstream of or within meiotic cell cycle and stem cell population maintenance.
P62962	Pfn1	This gene is involved in modification of postsynaptic actin cytoskeleton and synapse maturation. It acts upstream and within neural tube closure and regulation of transcription by RNA polymerase II.
Q3UEL5	Uroc1	This gene enables urocanate hydratase activity. It is involved in histidine catabolic processes. It can act upstream and within histidine metabolic processes and is found within cytosol.
Q9DAW9	Cnn3	This gene enables microtubule binding activity and is involved in negative regulation of ATPase activity. It is active in the cytoskeleton.
Q7TSJ2	Map6	This gene enables microtubule binding activity. It is predicted to be involved in axonal transport of mitochondrion.
Q60759	Gcdh	This gene enables activities such as that of fatty-acyl-CoA binding, flavin adenine dinucleotide binding, and glutaryl-CoA dehydrogenase. It is involved in fatty acid beta-oxidation using acyl-CoA dehydrogenase and fatty-acyl-CoA biosynthetic process. It is located in the mitochondrial inner membrane.
Q8K354	Cbr3	This gene enables activities such as 3-keto sterol reductase, NADPH binding, and carbonyl reductase (NADPH) activities. It is located in cytosol.
Q9CZR2	Naalad2	This protein enables N-formylglutamate deformylase activity and carboxypeptidase activity and is an integral component of membranes.
F6RBR6	Ndufa12	This gene is involved in mitochondrial ATP synthesis coupled electron transport and response to oxidative stress. It is located in mitochondrion.
Q9CYN2	Spcs2	This gene enables peptidase activity. Similarly, it can be involved in protein targeting to ER and signal peptide processing. It acts upstream of or within proteolysis. It is located in the endoplasmic reticulum and membrane.
E9QJR0	Lvrn	This gene enables activities such as metalloaminopeptidase, peptide binding, and zinc ion binding. It is predicted to be an integral component of the membrane.

*The biological functions were extracted from NCBI gene database (https://www.ncbi.nlm.nih.gov/gene).

Table S15. List of the top 10 upregulated and downregulated proteins in P-vEV and P-CRiSP-EV samples and their biological processes.

Accession	Gene ID	Biological Function*
A0A075B5R1	Ighv5-17	This gene enables both antigen and immunoglobulin receptor binding activities. It is involved in activation of immune response, defense response to other organism, and phagocytosis. It is predicted to be part of immunoglobulin complex.
O35326	Srsf5	The protein encoded by this gene is a member of the serine/arginine (SR)-rich family of pre-mRNA splicing factors, which constitute part of the spliceosome.
Q60759	Gcdh	This gene enables activities such as fatty-acyl-CoA binding, flavin adenine dinucleotide binding, and glutaryl-CoA dehydrogenase. It is involved in fatty acid beta-oxidation using acyl-CoA dehydrogenase and fatty-acyl-CoA biosynthetic process. It is located in mitochondrial inner membrane.
Q9JMH9	Myo18a	This gene enables ADP binding activity, ATP binding activity, and actin filament binding activity. It is involved in positive regulation of opsonization and regulation of macrophage activation. Located in brush border, cytoskeleton, and phagocytic vesicle.
Q8BVF2	Pdcl3	This gene enables vascular endothelial growth factor receptor 2 binding activity. Additionally, it is involved in angiogenesis and positive regulation of gene expression.
Q9D787	Ppil2	This gene enables peptidyl-prolyl cis-trans isomerase activity and ubiquitin-ubiquitin ligase activity. It is involved in protein localization to plasma membrane, protein peptidyl-prolyl isomerization, and protein polyubiquitination.
Q8R3S2	Tspan33	This gene encodes a member of the tetraspanin family which typically have four transmembrane domains. The encoded protein may be involved in the regulation of erythropoiesis.
Q8R084	Ugt2b1	This gene enables glucuronosyltransferase activity. It is involved in biphenyl catabolic process, cellular glucuronidation, and estrogen metabolic process. This gene is located in the endoplasmic reticulum and nuclear outer membrane.
Q8BI84	Mia3	This gene enables cargo receptor activity. It is located in endoplasmic reticulum membrane.

P46978	Stt3a	This gene enables dolichyl-diphosphooligosaccharide-protein glycotransferase activity. It is involved in co-translational protein modification, post-translational protein modification, and protein N-linked glycosylation via asparagine.
Q8VCR8	Mylk2	This gene enables myosin light chain binding activity and myosin light chain kinase activity. it is Involved in skeletal muscle satellite cell differentiation.
Q91Z83	Myh7	This gene enables several activities such as ATP binding, ATP hydrolysis, and identical protein binding. It acts upstream of or within cardiac muscle hypertrophy in response to stress and transition between fast and slow fiber.
A0A075B5S9	Ighv9-4	This gene enables both antigen and immunoglobulin receptor binding activities. It is involved in several processes, including activation of immune response, and phagocytosis.
O35864	Cops5	This gene enables enzyme binding activity and macrophage migration inhibitory factor binding activity. Acts upstream and within regulation of cell cycle. The gene is part of COP9 signalosome.
Q80VP1	Epn1	This gene enables activities such as clathrin adaptor, phospholipid binding, and transmembrane transporter binding. It is located in cytosol, nucleus, and pre-synapse. It is predicted to be extrinsic component of the following membranes: cytoplasmic side of plasma, postsynaptic, and presynaptic.
Q8VCN5	Cth	This gene is involved in the positive regulation of both I-kappaB kinase/NF-kappaB signaling and NF-kappaB transcription factor activity. It acts upstream of negative regulation of apoptotic signaling pathway. This gene is located in cytosol and active in cytoplasm.
P19096	Fasn	This gene enables fatty acid synthase activity. It acts upstream of or within cellular response to interleukin-4, epithelial cell development, and fatty acid biosynthetic process.
O09161	Casq2	This gene enables calcium ion binding activity. It is involved in regulation of heart rate, regulation of ion transmembrane transport, and sarcomere organization. This gene is part of junctional membrane complex.
P34928	Apoc1	This gene encodes a precursor plasma protein that is cleaved to yield a signal peptide and two alternatively processed mature peptides. This protein binds to free fatty acids preventing their uptake by cells.
E9Q735	Ube4a	The gene enables ubiquitin-ubiquitin ligase activity. It acts upstream and within protein polyubiquitination and is located in cytoplasm.
Q9Z180	Setbp1	This gene enables DNA binding activity and be located in cytosol and nuclear body.
Q99K95	Rtf2	This gene enables DNA binding activity.
Q5GLZ0	Cyp2c66	This gene enables several activities such as caffeine oxidase, heme binding, and monooxygenase. It is active in cytoplasm and intracellular membrane-bounded organelle.

Q61646	Hp	This gene encodes a plasma glycoprotein called haptoglobin that binds free hemoglobin. The encoded preproprotein undergoes proteolytic processing to generate alpha and beta subunits that form a disulfide-linked tetrameric protein that plays an important role in the sequestration and clearance of extracorpuscular hemoglobin. Mice lacking the encoded protein exhibit stunted development of lymphoid organs associated with lower counts of mature T and B cells in the blood and secondary lymphoid compartments.
P50228	Cxcl5	This gene encodes a protein that is a member of the CXC subfamily of chemokines. Chemokines recruit and activate leukocytes and are classified by function (inflammatory or homeostatic) or by structure. This protein is proposed to bind the G-protein coupled receptor chemokine (C-X-C motif) receptor 2 to recruit neutrophils and to have homeostatic and inflammatory functions. In mice, deficiency of this gene is associated with increased lung inflammation that is neutrophil-dependent.
Q8K2B3	Sdha	This gene enables activities such as electron transfer, flavin adenine dinucleotide binding, and succinate dehydrogenase (ubiquinone). It is involved in mitochondrial electron transport, succinate to ubiquinone, nervous system development, and succinate metabolic process.
Q9EQ32	Pik3ap1	This gene enables both identical protein and phosphatidylinositol 3-kinase regulatory subunit binding activities. It is involved in regulation of inflammatory response, regulation of signal transduction, and toll-like receptor signaling pathway. It is located in cytosol and plasma membrane.
Q8K2V6	Ipo11	This gene enables nuclear import signal receptor activity. It acts upstream and within ribosomal protein import into nucleus. This gene is active in cytosol and nuclear envelope.
D3YW09	Sf3a2	This gene is involved in U2-type prespliceosome assembly and positive regulation of neuron projection development. It acts upstream and within RNA splicing, via transesterification reactions.
Q6PHQ8	Naa35	This gene is involved in smooth muscle cell proliferation. It is located in cytoplasm.
Q3U9G9	Lbr	This gene enables delta14-sterol reductase activity. It is involved in cholesterol biosynthetic process and neutrophil differentiation and is located in nuclear membrane.
P0DOV2	Ifi204	This gene enables double-stranded DNA binding activity and transcription coregulator activity. Additionally, it is involved in cellular response to interferon-alpha.
P97467	Pam	This gene enables several activities such as metal ion binding, peptidylamidoglycolate lyase, and peptidylglycine monooxygenase. It is involved in several processes, including fatty acid primary amide biosynthetic process, peptide amidation, and response to zinc ion.
Q60953	Pml	This gene enables SMAD binding activity. It is involved in several processes, including defense response to other organism, regulation of gene expression, and signal transduction by p53 class mediator. It acts upstream and within several processes, including apoptotic signaling pathway, positive regulation of macromolecule metabolic process, and transmembrane receptor protein

		serine/threonine kinase signaling pathway. This gene is located in PML body, cytosol, and nuclear matrix and is an extrinsic component of endoplasmic reticulum membrane.
A0A1D5RMM8	Pxn	This gene enables activities such as BH4 domain binding, MAP-kinase scaffold, and neuropilin binding. It is involved in positive regulation of angiogenesis and transforming growth factor beta receptor signaling pathway. It acts upstream and within several processes, including focal adhesion assembly, integrin-mediated signaling pathway, and substrate adhesion-dependent cell spreading. Additionally, it is located in focal adhesion and lamellipodium.
Q8VE88	Fam114a2	This gene is expressed in several structures, including alimentary system, brain, genitourinary system, respiratory system, and sensory organ.

* The biological functions were extracted from NCBI gene database (https://www.ncbi.nlm.nih.gov/gene).
